# Ameliorating effect of probiotics in a rat model of chronic kidney disease

**DOI:** 10.1371/journal.pone.0281745

**Published:** 2023-03-30

**Authors:** Takio Inatomi, Mihoko Honma

**Affiliations:** 1 Inatomi Animal Hospital, Ota-ku, Tokyo, Japan; 2 Kusama Animal Health Laboratory, Kashima-shi, Saga, Japan; University of Jeddah, SAUDI ARABIA

## Abstract

Chronic kidney disease is a prevalent and significant disease worldwide. This study investigated the effects of a medicinal probiotic (BIO-THREE, TOA Biopharma Co., Ltd, Tokyo, Japan) with safety assurance that contained *Bacillus subtilis* TO-A, *Enterococcus faecium* T-110, and *Clostridium butyricum* TO-A in chronic kidney disease. BIO-THREE was approved as a medical drug by the Japanese Ministry of Health, Labour and Welfare and is widely used in the human medical field to improve various symptoms caused by abnormal intestinal microflora. Sixty male rats were randomly assigned to three groups: (1) normal group (n = 20, group 1), rats were given a normal diet for 3 weeks, followed by phosphate-buffered solution (once daily, orally) and a normal diet for 4 weeks; (2) control group (n = 20, Group 2), rats were given a normal diet including 0.75% adenine for 3 weeks, followed by phosphate-buffered saline (once daily, orally) and a normal diet for 4 weeks; and (3) probiotic group (n = 20, Group 3), rats were given a normal diet including 0.75% adenine for 3 weeks, followed by probiotics (once daily, orally) and a normal diet for 4 weeks. Probiotic administration resulted in a decrease in intestinal pH by increasing short-chain fatty acid (SCFA) production, and consequently suppressed the production of urea toxin production, thus, protecting renal function. The lower intestinal pH also promoted a reduction in the blood phosphorus levels by promoting ionisation of calcium and its binding to free phosphorus. This probiotic-induced increase in SCFA production reduced intestinal permeability, inhibited blood lipopolysaccharide and urea toxin production, and prevented the weakening of muscle function and strength. Moreover, it improved dysbiosis in the gut. This study shows the potential of this probiotics approved as medicinal drug to reduce chronic kidney disease progression, especially where safety is required. Further studies are warranted to validate these findings in humans.

## Introduction

Various metabolic, immunologic, protective, and other roles performed by the human gut microbiota affect human health [1]. Additionally, the gut microbiota can biosynthesise and convert substances that are essential for them and their host physiologically [2, 3]. The onset and development of specific human and animal diseases have an impact on the kind of microorganisms present in them and their quantity [4, 5]. With an estimated incidence rate of up to 16% [6], chronic kidney disease (CKD) is a prevalent and significant disease worldwide. End-stage renal disease can develop from CKD and only be treated by a kidney transplant or regular dialysis, both of which are expensive and may not be globally accessible [7]. The composition and number of gut bacteria are influenced by CKD [8, 9]. Gut microbiota is crucial to the pathophysiology and progression of CKD, which can cause direct or indirect damage to the gastrointestinal tract. For instance, uremia is known to alter the intestinal walls, increase intestinal permeability, which can further decrease intestinal barrier integrity and translocate bacterial components into the blood and, therefore, affect the immune system through continuous oxidative stress and systemic inflammation [10–12]. In contrast, intestinal dysbiosis linked to CKD can affect the synthesis of a range of metabolites, including a reduction in beneficial substances, such as short-chain fatty acids (SCFA), and an elevation in harmful uremic toxins [13]. As a result of this imbalance, uremic toxins, such as indoxyl sulphate (IS) and p-cresyl sulfate (pCS), accumulate and play a role in oxidative stress, systemic inflammation, and the fibrotic pathways of both renal and non-renal tissues [14–16]. Furthermore, these toxins are also known to affect the skeletal muscles [17]. CKD can also complicate sarcopenia [18]. The CKD stage is associated with an increased prevalence of sarcopenia [19], and the presence of sarcopenia in patients with CKD is associated with poor clinical outcomes [19]. Low skeletal muscle mass is associated with major adverse cardiovascular events in patients with CKD [19].

In probiotics, living bacteria are present in sufficient numbers to penetrate the digestive tract in their active form and provide beneficial effects [20, 21]. Probiotics may repair damage triggered by circumstances, such as the disruption of the gut microbiota owing to the use of antibiotics and/or illness. The effectiveness of probiotics in decreasing uremic toxin generation and restoring renal function has previously been investigated *in vitro* and in both animals and humans [22]. Because of their numerous health-improving benefits and innate ability to prevent certain illnesses, including CKD, administration of probiotics is considered a natural biotreatment. As many of the multifunctional biological functions of probiotics are very strain specific, not all probiotic strains are useful in every circumstance. Therefore, careful selection of organisms based on the desired therapeutic outcome is crucial [22]. Additionally, when probiotics are used by patients with a disease, safety is a significant factor. At present, only a few probiotics have been approved for clinical use based on a long-term experience of administration to humans with guaranteed safety.

Probiotics, such as *Bacillus subtilis* TO-A, *Enterococcus faecium* T-110 (TP1240), and *Clostridium butyricum* TO-A, are often used for the treatment and prevention of infectious diseases in Japan, China, and India. These microorganisms naturally lack genes related to virulence and pathogenesis [23–25] and have been administered to infants with Hirschsprung’s disease, patients with cancer, and pregnant women [26–29]. Furthermore, based on safety aspects, also considered suitable for CKD.

One-kidney one-clip [30], two-kidney one-clip [31], streptozotocin-induced diabetes [32], unilateral ureteral obstruction [33], and genetic models of diabetes and diabetic nephropathy, including the diabetic obese db/db mouse, obese ob/ob mouse, hypoinsulinaemic non-obese diabetic mouse, KKAy mouse, and New Zealand obese mouse [34, 35], have all been developed as animal models to study the causes and therapeutic interventions of CKD in humans. Since many animals do not develop CKD-associated cardiovascular disease, the majority of animal models do not accurately represent the complexity of human disease [36]. One exception is the rodent CKD diet model using adenine. Similar to CKD in humans, the chronic adenine diet models enable characterisation of renal and cardiovascular disease that is comparatively stable [36]. Interventions for reversal can also be investigated [36]. Furthermore, the adenine-induced CKD rat model is a common experimentally induced model for the development of CKD [37].

This study aimed to examine the effect of medical probiotics on body weight; serum chemistry parameters; uremic toxins; bacterial translocation; intestinal SCFA production; intestinal pH, microflora, and permeability; and muscle strength and function in an animal CKD model established by feeding adenine to animals [38], for the potential generalisation of such data for research on humans.

Previously, to the best of out knowledge, only a few studies have examined the effects of medical probiotics on muscle strength and function in CKD rat models.

## Materials and methods

The Ethics Committee of the Kusama Animal Health Laboratory in Kashima, Japan authorised all experimental and animal care practices (approval number: 2020–8). All experimental and animal care procedures were conducted in accordance with the fundamental guidelines for the proper conduct of animal experiments and related activities at academic research institutions under the jurisdiction of the Ministry of Education, Culture, Sports, Science, and Technology by the National Institutes of Health Guide for the Care and Use of Laboratory Animals. According to the ARRIVE guidelines, all experimental and animal care methods were reported.

### Experimental animals

Wistar/ST (9-week-old) male rats were purchased from Japan SLC, Inc. (Shizuoka, Japan). The animals were kept separately in an animal facility under tightly controlled conditions, including unlimited access to food and water (CE-2, CLEA Japan, Inc., Tokyo, Japan), a temperature of 23±2°C, and a relative humidity of 55±10% during a 12-h light/dark cycle.

CKD induction by adenine was performed using the methodology described previously [39]. After 1 week of acclimation, the rats were randomly assigned into the next three groups: (1) normal group (n = 20, Group 1), a normal diet was given to rats for 3 weeks, followed by phosphate-buffered solution (PBS) (FUJIFILM Wako Pure Chemical Corporation., Osaka, Japan) (once daily, orally) and a normal diet for 4 weeks; (2) control group (n = 20, Group 2), a normal diet containing 0.75% adenine (FUJIFILM Wako Pure Chemical Corporation) was given to rats for 3 weeks, followed by PBS (once daily, orally) and a normal diet for 4 weeks; and (3) probiotic group (n = 20, Group 3), a normal diet containing 0.75% adenine was given to rats for 3 weeks, followed by probiotics (once daily, orally) and a normal diet for 4 weeks.

The probiotics used in this study contained *B*. *subtilis* TO-A (5.0 × 10⁷ CFU g^-1^), *E*. *faecium* T-110 (2.0 × 10^8^ CFU g^-1^), and *C*. *butyricum* TO-A (5.0 × 10^7^ CFU g^-1^). The probiotics were dissolved in PBS to a final concentration of 12.5 mg/mL. In Group 3, the probiotic solution (4 mL/kg body weight) was orally administered. The rats in Group 3 received 50 mg/kg of probiotics. The probiotics used in the study were purchased from TOA Biopharma Co., Ltd. (Tokyo, Japan).

After study completion, the rats were euthanised by intraperitoneal administration of secobarbital sodium (150 mg/kg) (IONAL SODIUM for Injection, Nichi-Iko Pharmaceutical Co., Ltd., Tokyo, Japan).

### Sample collection

Blood samples were collected from the tail vein before study initiation (Week -3), 3 weeks after the start of the study (Week 0), and on the last day of the study (Week 4). To minimise the pain caused to the rats, blood was collected quickly from the tail vein using a rat retainer (CL-4904, CLEA Japan, Inc.). For serum and plasma collection, blood collection tubes (Capiject II, Terumo, Tokyo, Japan) were used. Blood samples were centrifuged at 3,000×g for 10 min at 4°C (Model 3700, Kubota Corp., Tokyo, Japan) to separate the serum and plasma. Serum samples were used to determine the creatinine (Cre), blood urea nitrogen (BUN), calcium (Ca), phosphorus (P) and uremic toxin levels, while the soluble CD14 (sCD14) levels were determined using plasma.

Faecal samples were also obtained at Weeks -3, 0, and 4 and were used for determining faecal pH, SCFA concentration, and bacterial species.

### Serum creatinine (Cre), blood urea nitrogen (BUN), calcium (Ca), and phosphorus (P) concentrations

Serum BUN, creatinine, calcium, and phosphorus were measured using a Hitachi 7180 automatic analyser (Hitachi 7180, Hitachi High-Technologies Corp., Tokyo, Japan).

### Serum concentration of uremic toxins

Serum concentrations of seven uremic toxins (hipuric acid, 3-carboxy-4-methyl-5-propyl-2-furan propionate [CMPF], indole-3-acetic acid [IAA], IS, pCS, para-cresyl glucuronide [pCG], trimethylamine N-oxide [TMAO]) were measured as reported previously [40]. Briefly, all serum samples were analysed on a Prominence HPLC system (Shimadzu, Kyoto, Japan) coupled with a 3200 QTRAP tandem mass spectrometer (Sciex, Les Ulis, France). A CMB-20A control module, a CTO-20AC column oven, a SIL-20AC XR autosampler, three LC-20A binary pumps, and a DGU-20A5 degasser constitute the chromatograph chain. A Turbo V ion source in electrospray ionisation (ESI) mode was attached to the tandem mass spectrometer. After preconditioning, samples were injected twice into the chromatography system (injection volume: 15 μL) and analysed in positive (for TMAO, IAA) or negative (for CMPF, HA, IS, pCS, pCG) modes. Positive and negative mode acquisitions were 2 and 2.5 min, respectively. Using an ultra-pentafluorophenyl (PFP) propyl pre-column (5 μm, 50×2.1 mm, Restek, Lisses, France) on an ultra-PFP propyl column (5 μm, 50×2.1 mm, Restek), chromatographic separation was performed at 40°C for both acquisition methods. A gradient of ultra-pure water with 0.1% formic acid (FUJIFILM Wako Pure Chemical Corporation) (mobile phase A) and acetonitrile with 0.1% formic acid (mobile phase B) provided at a flow rate of 0.8 mL/min was used to elute the column. Elution was conducted under the following conditions before acquisition in the negative mode: 0 min: 40% mobile phase B (B); 0–1 min: 40–80% B; 1–2 min: 80% B; 2–2.2 min: 80–40% B; and 2.2–2.5 min: 40% B. Elution was conducted under the following conditions before acquisition in the positive mode: 0–0.5 min: 10% B; 0.5–0.7 min: 10–80%; 0.7–1.5 min: 80%; 1.5–1.7 min: 80–10%; and 1.7–2 min: 10%. Data were collected in multiple reaction monitoring (MRM) mode following ionisation in negative or positive ESI mode. The following source parameters were used: ESI voltage, -4,500 V in the negative mode and 4,500 V in the positive mode; ion source temperature, 350°C; heater gas, 70 psi; nebuliser gas, 40 psi; and curtain gas, 30 psi. For each analyte, the spectrometer’s MRM transitions, declustering potential, entry potential, collision energy, and collision cell exit potential parameters were optimised. AnalystTM software version 1.6.2 (Sciex) was used for data collection and analysis.

### Plasma sCD14 concentration

The plasma sCD14 concentration was measured by the method described by Poesen et al [41]. In this study, a commercial enzyme-linked immunosorbent assay (ELISA) kit was used to analyse plasma sCD14 quantities (Rat sCD14 ELISA Kit, Seattle, WA, USA). The ELISA process was conducted in accordance with the manufacturer’s instructions.

### Faecal SCFA concentration

SCFA concentrations in faeces were measured using the method described previously [42]. The concentrations of both individual SCFA components (acetic acid and butyric acid) and total SCFA were measured. A micro-centrifuge tube containing 1 mL of 10% meta-phosphoric acid was filled with 0.5 g of faecal samples (FUJIFILM Wako Pure Chemical Corporation), with an internal standard of 0.4 μL of 4-methyl valeric acid (FUJIFILM Wako Pure Chemical Corporation) added per mL. The solution was mixed enough with a vortex mixer and centrifuged at 5,700 × g for 20 min at 4°C (Model 3520, Kubota Corp.). An HP Agilent 6890 series gas chromatograph coupled with an HP 5973 series mass spectrometer (Agilent Technologies Inc., Santa Clara, CA, USA) was used to determine the SCFA concentration of the supernatant. The columns were HP-free fatty acid polyester stationary phase capillary columns of polyethylene glycol on Shimalite TPA 60/80, measuring 30 m in length, with an internal diameter of 0.25 mm (Agilent Technologies). The conditions were as follows: 1 mL injection volume, 240°C injector temperature, and 12.15 psi pressure, with 1.1 mL/min constant flow and helium carrier. The oven programme was conducted under the following conditions: initial temperature of 80°C for 5 min followed by an increase of 10°C every min to 240°C for 12 min. The concentration of SCFA was given as μmol per g of wet faeces.

### pH of faeces

The pH of the faeces was measured by the method described by Kieffer et al [43]. In a clean tube, 400 mg of faeces were transferred. A 10:1 ratio of HPLC-grade water was added (Model 3700, Kubota Corp.). A Geno/Grinder (SPEX Sample Prep LLC, Metuchen, NJ, USA) was used to homogenise the contents for 2 min at 1,200 rpm before centrifugation at 3,509×g for 10 min at 4°C. To measure the pH of the faecal water, a pH meter (F70-S, HORIBA Advanced Techno, Co., Ltd, Kyoto, Japan) was used.

### Bacterial populations in faeces

Bacterial populations in faeces were determined by the FISH (fluorescence in situ hybridisation) method, as described by Martín-Peláez et al [44]. Faeces were directly placed in a clean microtube and mixed with 10% (w/v) of 0.1 M PBS at a pH of 7.4. For 2 min, the slurry was mixed and filtered in the stomacher bag. Thereafter, 500 μL of faecal samples were fixed for 4 h at 4°C in three volumes of ice-cold 4% (w/v) paraformaldehyde (FUJIFILM Wako Pure Chemical Corporation), which was then centrifuged at 13,000 g for 5 min and washed twice in 1 mL of sterile PBS. The cells were centrifuged into pellets and, then, resuspended in 150 μL of sterile PBS, which was mixed with 150 μL of ethanol (Fujifilm Wako Pure Chemical Corporation). The samples were vortexed and kept at -20°C until utilisation for hybridisation. For the hybridisations, six-well slides with a 10-mm diameter were covered with Teflon and polylysine, and 20 μL of each sample was pipetted onto each slide (Tekdon Inc., Myakka City, FL, USA). The samples were dried on the slides for 15 min at 46°C before being dehydrated in a series of alcohols (50%, 80%, and 96%) for 3 min each. After evaporating the ethanol from the slide, the probe was applied to the sample. To permeabilise the cells for use with probes Lab158 and Rfla729/Rbro730, the samples were treated with 50 μL of lysozyme (1 mg mL^−1^ in 100 mM Tris–HCl, pH 8.0) (FUJIFILM Wako Pure Chemical Corporation) at 37°C for 15 min before being washed briefly (2–3 s) in water followed by dehydration in a series of ethanol. On the surface of each well, a combination of probe and hybridisation buffer (5 μL of a 50 ng μL^-1^ stock of probe and 45 μL of hybridisation buffer) was applied. In an ISO20 oven, hybridisations were conducted for 4 h (Boekel Scientific, Feasterville-Trevos, PA, USA). The slides were washed in 50 mL of wash buffer containing 20 μL of 4’,6-diamidino-2-phenylindole dihydrochloride (DAPI; 50 ng L1; Sigma, ST Louis, MO, USA) for 15 min. The next step was a quick wash (2–3 s) in ice-cold water and drying with compressed air. Then, each well was filled with 5 μL of antifade reagent (polyvinyl alcohol mounting medium with DABCOTM antifading; Sigma, Tokyo, Japan) before a coverslip was applied on top of each of them. Until the cells were counted using a Nikon E400 Eclipse microscope (Nikon, Tokyo, Japan), the slides were kept in the dark at 4°C (for a maximum of 3 days). The DM 400 and DM 575 filters were used to visualise DAPI-stained slides and to probe slides, respectively. The following equation was used to calculate the numbers of certain bacteria and DAPI-stained objects (used to count all bacteria):

$$DF\times ACC\times 6732.42\times 50\times DF_{sample},$$

Here, DF is the dilution factor (300/500 = 0.6), ACC is the average cell count over 15 fields of vision, and DFsample is the sample dilution employed with a specific probe or stain (for example, 50 for Bif164 counts). The area of the well divided by the area of the field of vision is represented by the figure 6732.42, and the factor 50 denotes the cell count per mL of sample. The amount of each bacterium was expressed per gram of wet faeces after the units were changed from per mL to per gram. All the probes were created and labelled with Cy3 by Sigma-Aldrich Japan (Tokyo, Japan). The details of the probes used in this study are shown in Table 1 [45–48].

**Table 1:** FISH probes for bacterial population analysis in faeces.

Short name	Accession no.	Full name	Target species	Temperature (°C)		Sequence (5′ to 3′)	Reference
				Hybridisation	Washing		
**Bif164**	pB-00037	S-G-Bif-0164-a-A-18	Most *Bifidobacterium* spp. and *Parascardovia denticolens*	50	50	CATCCGGCATTACCACCC	Langendijk et al. (1995) [45]
**Lab158**	ND	S-G-Lab-0158-a-A-20	Most *Lactobacillus*, *Leuconostoc*, and *Weissella* spp.; *Lactococcus lactis*; all *Vagococcus*, *Enterococcus*, *Melisococcus*, *Tetragenococcus*, *Catellicoccus*, *Pediococcus*, and *Paralactobacillus* spp.	50	50	GGTATTAGCAYCTGTTTCCA	Harmsen et al. (1999) [46]
**Erec482**	pB-00963	S-* -Erec0482-a-A-19	Most members of Clostridium cluster XIVa; *Syntrophococcus sucromutans*, *Bacteroides galacturonicus* and *Bacteroides xylanolyticus*, *Lachnospira pectinschiza*, and *Clostridium saccharolyticum*	50	50	GCTTCTTAGTCARGTACCG	Franks et al. (1998) [47]
**EC 1531**	pB-3938	L-S-Eco-1531-a-A-21	*E*. *coli* spp.	37	37	CACCGTAGTGCCTCGTCATCA (23S rRNA)	Poulsen et al. (1994) [48]

*ND, There is nothing in probeBase (http://www.microbial-ecology.net/probebase) concerning these probes.

Probe designation by Alm et al. (1996) [49]. This information was gathered from probeBase.

Both probes were used in combination at equimolar doses (50 ng μL^-1^).

The hybridisation buffer included 20% formamide.

### Intestinal permeability

Intestinal permeability was evaluated using the method described by Cani et al. [50] on the final day (Week 4) of either the probiotic-including or normal diet. The evaluation was based on the intestinal permeability to 4,000-Da fluorescent dextran (Sigma-Aldrich), as previously described [51]. Rats were given fluorescein isothiocyanate (FITC)-dextran (600 mg/kg body weight, 125 mg/mL) orally after fasting for 6 h. After 1 h, 120 μL of blood was collected from the tail vein, which was centrifuged at 12,000×g for 3 min at 4°C. The concentration of FITC-dextran in plasma was measured using a fluorescence spectrophotometer (HTS-7000 Plus-plate-reader; Perkin Elmer Japan Co., Ltd., Yokohama, Japan) after it was diluted in an equivalent volume of PBS (pH 7.4). Standard curves for calculating the concentration of FITC-dextran in the samples were made by diluting FITC-dextran in non-treated plasma that had been diluted with PBS (1:2 [v/v]).

### Muscle strength

To gauge muscular strength, a grip force metre (MK-380CM/R, Muromachi Kikai, Tokyo, Japan) was used to quantify forelimb grip force [52]. This measurement was made using a force gauge that was mounted on the device’s front. Each rat was held during the test while its tail was gently moved from rostral to caudal direction, imparting force to the mesh grid. The rat was placed with both forepaws inside the front grip grid after the gauge had been zeroed. The rat was pulled steadily backward by its tail once it had a firm grip on the grid until it lost it. Three successive measurements were obtained after taking note of the gauge’s reading (N), zeroing the strain gauge, and testing the rat once again. Based on the force produced, comparisons between the different animal groups were made.

### Muscle function

Muscle function was assessed as motor performance and balance and was evaluated using a rotarod (LE8205, Panlab, Barcelona, Spain), as described previously [53]. Individual rats were trained with a rotor rod at a constant speed (4 rpm) for 5 days (once/day) prior to the test to ensure stable performance. On the day of the test, the rats were placed on a rotor rod and evaluated for time until they fell over in acceleration mode (4–40 rpm for 60 s). Each rat was tested five times, with a few minutes of recovery time between the tests. The average value measured was used as the motor coordination value.

### Statistical analyses

The sample size was determined using Cohen’s method [54] (effect size = 0.4 and power = 0.8 and 0.05), and N was determined using G*Power (University of Dusseldorf, Dusseldorf, Germany) [55]. Multiple comparison tests were used to compare the groups. To check the homogeneity of the variance, the Bartlett test was used. When the Bartlett test did not detect any significant deviation, a one-way analysis of variance was performed to determine whether there were any differences in the means of the three groups. The significance of the intergroup mean differences was then examined using the Bonferroni’s test. A non-parametric comparison using the Kruskal–Wallis H test was performed if a deviation from variance homogeneity was significant. At a *p*-value <0.05, the results were considered significant. The statistical analyses were performed using EZR software (Saitama Medical Centre, Jichi Medical University, Saitama, Japan); EZR is a graphical user interface for R (version 2.13.0; The R Foundation for Statistical Computing, Vienna, Austria).

## Results

This study aimed to examine the effects of a medicinal probiotic with safety assurance on CKD. For this purpose, body weight, renal function, intestinal function, and muscle function were investigated in rats with CKD caused by adenine. In these rats, administration of the medical probiotic resulted in improvements in body weight, renal function, intestinal function, and muscle function. Based on these results, administration of this medical probiotic was considered safe and effective in patients with CKD.

### Body weights

Table 2 shows the body weights of the animals belonging to the three study groups. At Weeks 0 (between Groups 1 and 2 and Groups 1 and 3) and 4 (between Groups 1 and 2, Groups 1 and 3, and Groups 2 and 3), significant differences were detected.

**Table 2:** The effect of probiotics treatment on body weight (g) in adenine-induced CKD rats.

	-3 w	0 w	4 w
**Normal group**	310.0±2.627	375.7±4.086^a^	404.2±3.523^a^
**Control group**	313.7±3.063	251.7±2.144^b^	287.8±2.158^b^
**Probiotics group**	314.6±2.573	253.6±2.724^b^	304.2±2.197^c^

The normal group (N = 20) was untreated. The control group (N = 20) and the probiotics group (N = 20) received adenine orally from Week -3 to Week 0 to induce CKD. The control group received PBS orally from Week 0 to Week 4. The probiotics group received probiotics dissolved in PBS orally from Week 0 to Week 4. When the Bartlett test did not detect any significant deviation, a one-way analysis of variance was performed to determine whether there were any differences in the means of the three groups. Then, the significance of the intergroup mean differences was examined using Bonferroni’s test. A non-parametric comparison using the Kruskal–Wallis H test was performed if a deviation from variance homogeneity was significant. The level of significance was set at *p*<0.05. Data are presented as means ± standard errors.

^a, b, c^ Different letters within columns indicate differences between the treatment groups (*p*<0.05).

CKD, chronic kidney disease; PBS, phosphate-buffered solution

### Total SCFA and n-butyrate concentrations in faeces

Tables 3 and 4 show the total SCFA and n-butyrate concentrations in faeces, respectively. At Weeks 0 (between Groups 1 and 2 and Groups 1 and 3) and 4 (between Groups 1 and 2, Groups 1 and 3, and Groups 2 and 3), significant differences were detected.

**Table 3:** The effect of probiotics treatment on total short-chain fatty acid concentration in faeces (μmol/g) in adenine-induced CKD rats.

	-3 w	0 w	4 w
**Normal group**	63.1±1.75	61.4±2.03^a^	60.7±2.31^a^
**Control group**	58.3±2.05	41.1±1.19^b^	38.7±1.27^b^
**Probiotics group**	59.7±1.52	39.0±1.57^b^	50.8±1.14^c^

The normal group (N = 20) was untreated. The control group (N = 20) and the probiotics group (N = 20) received adenine orally from Week -3 to Week 0 to induce CKD. The control group received PBS orally from Week 0 to Week 4. The probiotics group received probiotics dissolved in PBS orally from Week 0 to Week 4. When the Bartlett test did not detect any significant deviation, a one-way analysis of variance was performed to determine whether there were any differences in the means of the three groups. Then, the significance of the intergroup mean differences was examined using Bonferroni’s test. A non-parametric comparison using the Kruskal–Wallis H test was performed if a deviation from variance homogeneity was significant. The level of significance was set at *p*<0.05. Data are presented as means ± standard errors.

^a, b, c^ Different letters within columns indicate differences between the treatment groups (*p*<0.05).

CKD, chronic kidney disease; PBS, phosphate-buffered solution

**Table 4:** The effect of probiotics treatment on N-butyrate concentration in faeces (μmol/g) in adenine-induced CKD rats.

	-3 w	0 w	4 w
**Normal group**	4.13±0.14	3.91±0.12^a^	4.01±0.14^a^
**Control group**	3.95±0.09	1.19±0.04^b^	0.97±0.05^b^
**Probiotics group**	4.03±0.13	1.15±0.06^b^	1.45±0.06^c^

The normal group (N = 20) was untreated. The control group (N = 20) and the probiotics group (N = 20) received adenine orally from Week -3 to Week 0 to induce CKD. The control group received PBS orally from Week 0 to Week 4. The probiotics group received probiotics dissolved in PBS orally from Week 0 to Week 4. When the Bartlett test did not detect any significant deviation, a one-way analysis of variance was performed to determine whether there were any differences in the means of the three groups. Then, the significance of the intergroup mean differences was examined using Bonferroni’s test. A non-parametric comparison using the Kruskal–Wallis H test was performed if a deviation from variance homogeneity was significant. The level of significance was set at *p*<0.05. Data are presented as means ± standard errors.

^a, b, c^ Different letters within columns indicate differences between the treatment groups (*p*<0.05).

CKD, chronic kidney disease; PBS, phosphate-buffered solution

### Acetate concentration

Table 5 shows acetate concentrations in faeces. At Week 0, no significant differences were observed in terms of acetate concentrations in faeces among the three groups, while at Week 4, faece acetate concentrations were significantly different between Groups 1 and 2.

**Table 5:** The effect of probiotics treatment on acetate concentration in faeces (μmol/g) in adenine-induced CKD rats.

	-3 w	0 w	4 w
**Normal group**	21.6±1.47	20.7±1.35	20.9±1.61^a^
**Control group**	20.8±1.39	17.8±1.12	13.8±1.18^b^
**Probiotics group**	19.1±0.96	16.7±1.02	18.4±1.55

The normal group (N = 20) was untreated. The control group (N = 20) and the probiotics group (N = 20) received adenine orally from Week -3 to Week 0 to induce CKD. The control group received PBS orally from Week 0 to Week 4. The probiotics group received probiotics dissolved in PBS orally from Week 0 to Week 4. When the Bartlett test did not detect any significant deviation, a one-way analysis of variance was performed to determine whether there were any differences in the means of the three groups. Then, the significance of the intergroup mean differences was examined using Bonferroni’s test. A non-parametric comparison using the Kruskal–Wallis H test was performed if a deviation from variance homogeneity was significant. The level of significance was set at *p*<0.05. Data are presented as means ± standard errors.

^a, b, c^ Different letters within columns indicate differences between the treatment groups (*p*<0.05).

CKD, chronic kidney disease; PBS, phosphate-buffered solution

### Intestinal pH, intestinal permeability, plasma sCD14 concentration, and serum uremic toxin concentrations

Significant differences in the intestinal pH, intestinal permeability, plasma sCD14 concentration, and serum uremic toxin concentrations were observed among the three groups at Weeks 0 (between Groups 1 and 2 and Groups 1 and 3) and 4 (between Groups 1 and 2, Groups 1 and 3, and Groups 2 and 3) (Tables 6–9).

**Table 6:** The effect of probiotics treatment on pH of faeces in adenine-induced CKD rats.

	-3 w	0 w	4 w
**Normal group**	6.33±0.22	5.86±0.11^a^	6.10±0.14^a^
**Control group**	5.92±0.16	7.47±0.10^b^	7.48±0.09^b^
**Probiotics group**	5.75±0.16	7.45±0.10^b^	6.55±0.09^c^

The normal group (N = 20) was untreated. The control group (N = 20) and the probiotics group (N = 20) received adenine orally from Week -3 to Week 0 to induce CKD. The control group received PBS orally from Week 0 to Week 4. The probiotics group received probiotics dissolved in PBS orally from Week 0 to Week 4. When the Bartlett test did not detect any significant deviation, a one-way analysis of variance was performed to determine whether there were any differences in the means of the three groups. Then, the significance of the intergroup mean differences was examined using Bonferroni’s test. A non-parametric comparison using the Kruskal–Wallis H test was performed if a deviation from variance homogeneity was significant. The level of significance was set at *p*<0.05. Data are presented as means ± standard errors.

^a, b, c^ Different letters within columns indicate differences between the treatment groups (*p*<0.05).

CKD, chronic kidney disease; PBS, phosphate-buffered solution

**Table 7:** The effect of probiotics treatment on intestinal permeability in adenine-induced CKD rats.

	-3 w	0 w	4 w
**Normal group**	0	0^a^	0^a^
**Control group**	0	0.298±0.014^b^	0.391±0.012^b^
**Probiotics group**	0	0.288±0.014^b^	0.132±0.013^c^

Data represent Plasma DX-4000-FITC (μg/mL). There is no detection of DX-4000-FITC in the blood without impaired intestinal permeability.

The normal group (N = 20) was untreated. The control group (N = 20) and the probiotics group (N = 20) received adenine orally from Week -3 to Week 0 to induce CKD. The control group received PBS orally from Week 0 to Week 4. The probiotics group received probiotics dissolved in PBS orally from Week 0 to Week 4. When the Bartlett test did not detect any significant deviation, a one-way analysis of variance was performed to determine whether there were any differences in the means of the three groups. Then, the significance of the intergroup mean differences was examined using Bonferroni’s test. A non-parametric comparison using the Kruskal–Wallis H test was performed if a deviation from variance homogeneity was significant. The level of significance was set at *p*<0.05. Data are presented as means ± standard errors.

^a, b, c^ Different letters within columns indicate differences between the treatment groups (*p*<0.05).

CKD, chronic kidney disease; PBS, phosphate-buffered solution

**Table 8:** The effect of probiotics treatment on plasma soluble CD14 concentrations (μg/mL) in adenine-induced CKD rats.

	-3 w	0 w	4 w
**Normal group**	1.95±0.06	1.97±0.06^a^	1.89±0.06^a^
**Control group**	2.11±0.06	4.44±0.18^b^	5.08±0.12^b^
**Probiotics group**	1.93±0.07	4.60±0.18^b^	3.10±0.10^c^

The normal group (N = 20) was untreated. The control group (N = 20) and the probiotics group (N = 20) received adenine orally from Week -3 to Week 0 to induce CKD. The control group received PBS orally from Week 0 to Week 4. The probiotics group received probiotics dissolved in PBS orally from Week 0 to Week 4. When the Bartlett test did not detect any significant deviation, a one-way analysis of variance was performed to determine whether there were any differences in the means of the three groups. Then, the significance of the intergroup mean differences was examined using Bonferroni’s test. A non-parametric comparison using the Kruskal–Wallis H test was performed if a deviation from variance homogeneity was significant. The level of significance was set at *p*<0.05. Data are presented as means ± standard errors.

^a, b, c^ Different letters within columns indicate differences between the treatment groups (*p*<0.05).

CKD, chronic kidney disease; PBS, phosphate-buffered solution

**Table 9:** The effect of probiotics treatment on serum uremic toxin concentration (μM) in adenine-induced CKD rats.

	**Week**	**Normal group**	**Control group**	**Probiotics group**
**Para-cresyl sulfate**	-3 w	9.709±0.714	10.05±0.567	9.327±0.581
	0 w	10.27±0.638^a^	100.0±2.912^b^	105.4±2.916^b^
	4 w	9.035±0.683^a^	101.0±2.141^b^	75.40±2.987^c^
**Para-cresyl glucuronide**	-3 w	0.058±0.002	0.053±0.001	0.052±0.002
	0 w	0.053±0.002^a^	0.500±0.017^b^	0.449±0.027^b^
	4 w	0.055±0.002^a^	0.631±0.030^b^	0.387±0.014^c^
**Indoxyl sulfate**	-3 w	2.052±0.059	1.993±0.074	2.021±0.062
	0 w	2.002±0.052^a^	69.02±3.109^b^	68.95±1.864^b^
	4 w	1.897±0.073^a^	70.17±3.051^b^	53.43±1.528^c^
**Indole-3-acetic acid**	-3 w	2.117±0.057	2.083±0.075	2.150±0.057
	0 w	2.066±0.065^a^	4.934±0.061^b^	4.973±0.073^b^
	4 w	2.049±0.056^a^	4.982±0.078^b^	2.981±0.030^c^
**Trimethylamine N-oxide**	-3 w	2.013±0.040	2.026±0.037	2.009±0.038
	0 w	1.970±0.043^a^	38.50±1.788^b^	40.81±2.019^b^
	4 w	2.013±0.040^a^	40.99±1.495^b^	28.44±0.970^c^
**CMPF (3-carboxy-4-methyl-5-propyl-2-furanpropionate)**	-3 w	1.999±0.040	1.934±0.041	2.037±0.049
	0 w	1.974±0.048^a^	44.26±1.070^b^	46.18±1.091^b^
	4 w	2.020±0.043^a^	45.83±1.050^b^	40.04±0.579^c^
**Hippuric acid**	-3 w	8.223±0.251	7.311±0.216	7.793±0.189
	0 w	7.796±0.249^a^	40.72±0.954^b^	40.92±1.448^b^
	4 w	7.979±0.178 ^a^	39.94±0.921^b^	29.68±0.666^c^

The normal group (N = 20) was untreated. The control group (N = 20) and the probiotics group (N = 20) received adenine orally from Week -3 to Week 0 to induce CKD. The control group received PBS orally from Week 0 to Week 4. The probiotics group received probiotics dissolved in PBS orally from Week 0 to Week 4. When the Bartlett test did not detect any significant deviation, a one-way analysis of variance was performed to determine whether there were any differences in the means of the three groups. Then, the significance of the intergroup mean differences was examined using Bonferroni’s test. A non-parametric comparison using the Kruskal–Wallis H test was performed if a deviation from variance homogeneity was significant. The level of significance was set at *p*<0.05. Data are presented as means ± standard errors.

^a, b, c^ Different letters within rows indicate differences between the treatment groups (*p*<0.05).

CKD, chronic kidney disease; PBS, phosphate-buffered solution

### Enumeration of bacterial populations in faeces using FISH

The amounts of *Bifidobacterium* sp., *Lactobacillus/Enterococcus* spp., *Clostridium coccoides-Eubacterium rectale* group, and *Escherichia*. *coli* spp. in the faeces are presented in Table 10. The amount of these bacterial populations was (1) significantly different between Groups 1 and 2 and Groups 1 and 3 at Week 0, and (2) significantly different between Groups 1 and 2 and Groups 2 and 3 at Week 4.

**Table 10:** The effect of probiotics treatment on microbiological analyses of faeces (log cells/g) in adenine-induced CKD rats.

	Week	Normal group	Control group	Probiotics group
**Lab158**	-3 w	10.0980.15	10.03±0.21	10.04±0.19
	0 w	10.03±0.15^a^	9.00±0.12^b^	9.28±0.15^b^
	4 w	10.00±0.21^a^	8.97±0.15^b^	9.46±0.06^a^
**Bif164**	-3 w	9.81±0.12	9.94±0.19	9.91±0.13
	0 w	10.02±0.19^a^	9.25±0.13^b^	9.18±0.11^b^
	4 w	10.34±0.20^a^	8.91±0.08^b^	9.65±0.19^a^
**Erec482**	-3 w	10.03±0.11	10.08±0.10	10.05±0.11
	0 w	10.19±0.16^a^	9,16±0.09^b^	9.26±0.11^b^
	4 w	10.10±0.15^a^	9.10±0.10^b^	9.76±0.07^a^
**EC 1531**	-3 w	9.26±0.07	9.36±0.07	9.29±0.07
	0 w	9.34±0.08^a^	9.83±0.06^b^	9.76±0.06^b^
	4 w	9.27±0.07^a^	9.84±0.06^b^	9.38±0.04^a^
**DAPI**	-3 w	11.53±0.04	11.46±0.04	11.45±0.04
	0 w	11.53±0.05	11.52±0.05	11.50±0.04
	4 w	11.49±0.04	11.46±0.04	11.47±0.03

The normal group (N = 20) was untreated. The control group (N = 20) and the probiotics group (N = 20) received adenine orally from Week -3 to Week 0 to induce CKD. The control group received PBS orally from Week 0 to Week 4. The probiotics group received probiotics dissolved in PBS orally from Week 0 to Week 4. When the Bartlett test did not detect any significant deviation, a one-way analysis of variance was performed to determine whether there were any differences in the means of the three groups. Then, the significance of the intergroup mean differences was examined using Bonferroni’s test. A non-parametric comparison using the Kruskal–Wallis H test was performed if a deviation from variance homogeneity was significant. The level of significance was set at *p*<0.05. Data are presented as means ± standard errors.

^a, b, c^ Different letters within rows indicate differences between the treatment groups (*p*<0.05).

CKD, chronic kidney disease; PBS, phosphate-buffered solution

### Serum BUN, Cre, Ca, and P concentrations

Tables 11–14 show the serum BUN, Cre, Ca, and P levels. Among the three groups, the serum Ca levels were not significantly different. At Week 0, the serum BUN, Cre, and P levels presented significant differences between Groups 1 and 2 and Groups 1 and 3. Further, at Week 4, the serum BUN, Cre, and P levels showed significant differences between Groups 1 and 2, Groups 1 and 3, and Groups 2 and 3.

**Table 11:** The effect of probiotics treatment on serum blood urea nitrogen concentration (mg/dL) in adenine-induced CKD rats.

	-3 w	0 w	4 w
**Normal group**	15.8±0.33	16.1±0.35^a^	15.6±0.39^a^
**Control group**	16.0±0.40	152.0±2.49^b^	137.7±2.99^b^
**Probiotics group**	16.3±0.38	161.1±3.78^b^	121.1±3.30^c^

The normal group (N = 20) was untreated. The control group (N = 20) and the probiotics group (N = 20) received adenine orally from Week -3 to Week 0 to induce CKD. The control group received PBS orally from Week 0 to Week 4. The probiotics group received probiotics dissolved in PBS orally from Week 0 to Week 4. When the Bartlett test did not detect any significant deviation, a one-way analysis of variance was performed to determine whether there were any differences in the means of the three groups. Then, the significance of the intergroup mean differences was examined using Bonferroni’s test. A non-parametric comparison using the Kruskal–Wallis H test was performed if a deviation from variance homogeneity was significant. The level of significance was set at *p*<0.05. Data are presented as means ± standard errors.

^a, b, c^ Different letters within columns indicate differences between the treatment groups (*p*<0.05).

CKD, chronic kidney disease; PBS, phosphate-buffered solution

**Table 12:** The effect of probiotics treatment on serum creatinine concentration (mg/dL) in adenine-induced CKD rats.

	-3 w	0 w	4 w
**Normal group**	0.49±0.01	0.51±0.01^a^	0.50±0.01^a^
**Control group**	0.50±0.01	2.16±0.05^b^	2.07±0.06^b^
**Probiotics group**	0.49±0.01	2.09±0.07^b^	1.39±0.06^c^

The normal group (N = 20) was untreated. The control group (N = 20) and the probiotics group (N = 20) received adenine orally from Week -3 to Week 0 to induce CKD. The control group received PBS orally from Week 0 to Week 4. The probiotics group received probiotics dissolved in PBS orally from Week 0 to Week 4. When the Bartlett test did not detect any significant deviation, a one-way analysis of variance was performed to determine whether there were any differences in the means of the three groups. Then, the significance of the intergroup mean differences was examined using Bonferroni’s test. A non-parametric comparison using the Kruskal–Wallis H test was performed if a deviation from variance homogeneity was significant. The level of significance was set at *p*<0.05. Data are presented as means ± standard errors.

^a, b, c^ Different letters within columns indicate differences between the treatment groups (*p*<0.05).

CKD, chronic kidney disease; PBS, phosphate-buffered solution

**Table 13:** The effect of probiotics treatment on serum calcium concentration (mg/dL) in adenine-induced CKD rats.

	-3 w	0 w	4 w
**Normal group**	9.52±0.12	9.89±0.15	9.44±0.11
**Control group**	9.55±0.11	9.66±0.12	9.65±0.10
**Probiotics group**	9.66±0.10	9.58±0.08	9.69±0.14

The normal group (N = 20) was untreated. The control group (N = 20) and the probiotics group (N = 20) received adenine orally from Week -3 to Week 0 to induce CKD. The control group received PBS orally from Week 0 to Week 4. The probiotics group received probiotics dissolved in PBS orally from Week 0 to Week 4. When the Bartlett test did not detect any significant deviation, a one-way analysis of variance was performed to determine whether there were any differences in the means of the three groups. Then, the significance of the intergroup mean differences was examined using Bonferroni’s test. A non-parametric comparison using the Kruskal–Wallis H test was performed if a deviation from variance homogeneity was significant. The level of significance was set at *p*<0.05. Data are presented as means ± standard errors.

**Table 14:** The effect of probiotics treatment on serum phosphorus concentration (mg/dL) in adenine-induced CKD rats.

	-3 w	0 w	4 w
**Normal group**	4.94±0.14	5.14±0.09^a^	4.74±0.11^a^
**Control group**	4.90±0.13	7.85±0.19^b^	8.16±0.27^b^
**Probiotics group**	4.72±0.13	7.55±0.17^b^	7.20±0.21^c^

The normal group (N = 20) was untreated. The control group (N = 20) and the probiotics group (N = 20) received adenine orally from Week -3 to Week 0 to induce CKD. The control group received PBS orally from Week 0 to Week 4. The probiotics group received probiotics dissolved in PBS orally from Week 0 to Week 4. When the Bartlett test did not detect any significant deviation, a one-way analysis of variance was performed to determine whether there were any differences in the means of the three groups. Then, the significance of the intergroup mean differences was examined using Bonferroni’s test. A non-parametric comparison using the Kruskal–Wallis H test was performed if a deviation from variance homogeneity was significant. The level of significance was set at *p*<0.05. Data are presented as means ± standard errors.

^a, b, c^ Different letters within columns indicate differences between the treatment groups (*p*<0.05).

CKD, chronic kidney disease; PBS, phosphate-buffered solution

### Muscle strength and function

Significant differences in the muscle strength and function were observed among the three groups at Weeks 0 (between Groups 1 and 2 and Groups 1 and 3) and 4 (between Groups 1 and 2, Groups 1 and 3, and Groups 2 and 3; Tables 15 and 16).

**Table 15:** The effect of probiotics treatment on muscle strength (Grip Force) (N) in adenine-induced CKD rats.

	-3 w	0 w	4 w
**Normal**	13.1±0.4	13.8±0.4^a^	14.7±0.5^a^
**Control**	12.3±0.5	10.7±0.4^b^	9.8±0.4^b^
**Probiotics**	12.6±0.5	11.7±0.4^b^	11.5±0.4^c^

The normal group (N = 20) was untreated. The control group (N = 20) and the probiotics group (N = 20) received adenine orally from Week -3 to Week 0 to induce CKD. The control group received PBS orally from Week 0 to Week 4. The probiotics group received probiotics dissolved in PBS orally from Week 0 to Week 4. When the Bartlett test did not detect any significant deviation, a one-way analysis of variance was performed to determine whether there were any differences in the means of the three groups. Then, the significance of the intergroup mean differences was examined using Bonferroni’s test. A non-parametric comparison using the Kruskal–Wallis H test was performed if a deviation from variance homogeneity was significant. The level of significance was set at *p*<0.05. Data are presented as means ± standard errors.

^a, b, c^ Different letters within columns indicate differences between the treatment groups (*p*<0.05).

CKD, chronic kidney disease; PBS, phosphate-buffered solution

**Table 16:** The effect of probiotics treatment on muscle function (Permanence time) (s) in adenine-induced CKD rats.

	-3 w	0 w	4 w
**Normal**	23.6±0.7	25.0±0.7^a^	28.5±0.5^a^
**Control**	23.7±1.2	19.7±0.6^b^	16.6±0.6^b^
**Probiotics**	23.0±0.7	20.4±0.7^b^	20.8±0.7^c^

The normal group (N = 20) was untreated. The control group (N = 20) and the probiotics group (N = 20) received adenine orally from Week -3 to Week 0 to induce CKD. The control group received PBS orally from Week 0 to Week 4. The probiotics group received probiotics dissolved in PBS orally from Week 0 to Week 4. When the Bartlett test did not detect any significant deviation, a one-way analysis of variance was performed to determine whether there were any differences in the means of the three groups. Then, the significance of the intergroup mean differences was examined using Bonferroni’s test. A non-parametric comparison using the Kruskal–Wallis H test was performed if a deviation from variance homogeneity was significant. The level of significance was set at *p*<0.05. Data are presented as means ± standard errors.

^a, b, c^ Different letters within columns indicate differences between the treatment groups (*p*<0.05).

CKD, chronic kidney disease; PBS, phosphate-buffered solution

## Discussion

CKD is a multifactorial disease [56, 57] related to health problems associated with reduced quality of life [58], high management costs, and increased risk of death. In advanced CKD, the intake of fruits and vegetables must be limited to prevent the risk of hyperkalaemia and fluid overload. This lack of fiber amplifies the predisposition to dysbiosis, including delayed intestinal transit, oedema of the intestinal wall, and increased metabolic acidosis [59, 60]. Many studies have been conducted using probiotics to ameliorate various symptoms resulting from CKD-induced alterations in the intestinal environment. However, most of these studies used supplements, and only few have used probiotics that have been approved by the regulatory authorities for medicinal drugs in the country or region and confirmed to be safe for medical use. Therefore, in this study, we investigated the effects of medical probiotics on the adenine-induced CKD rat model, with scope for future application to humans.

Yokozawa et al. [15] described the adenine-induced CKD rat model as a model for renal failure. Adenine taken orally is quickly converted to 2,8-dihydroxyadenine, which is then crystallised and deposited in the microvilli and apical domains of the epithelia in the proximal renal tubules, leading to the degeneration of the renal tubule and interstitium and the development of renal failure [16]. In previous studies [61–63], we also suggested that adenine-induced tubular dysfunction hindered renal clearance of uremic toxins, causing a rise in serum uremic toxins even after 4 weeks on a regular diet. Furthermore, it has been noted that adenine-induced CKD rats had increased intestinal permeability [64], which can also lead to an increase in toxins. In this study, we found that the intestinal permeability increased in the control and probiotics groups compared with the normal group at Week 0. Higher IS and pCS concentrations are known to cause exacerbation of tubular damage [37, 38]. This suggests that the high IS and pCS levels observed in this study promote CKD progression. Moreover, our results further confirmed the successful induction of CKD by adenine.

The doses of the probiotics in this study that contained *B*. *subtilis* TO-A, *E*. *faecium* T-110, and *C*. *butyricum* TO-A and were administered to rats, were determined based on the doses given to humans. Moreover, these doses administered to rats did not have negative side effects based on veterinary diagnosis.

A previous study [65] reported a decrease in faecal SCFA concentrations in adenine-induced CKD rats due to CKD-induced changes in the gut microbiota. Regarding butyric acid, among the faecal SCFAs, adenine treatment significantly decreased its levels both in a previous study [65] and in the present study. Regarding acetic acid, there was a non-significant reduction in both the current and previous studies [65]. Propionic acid was reduced but not significantly in this study (data not shown). In contrast, it was significantly reduced in a previous study [65]. The reasons for the different results between the previous [65] and the present study regarding the effect of adenine administration on faecal propionic acid concentrations remain unclear and require further research. In this study, the probiotics increased faecal butyrate concentrations in rats with CKD caused by adenine. Probiotics can increase SCFA production in humans and animals [66]. Moreover, probiotics, including *B*. *subtilis* TO-A, *E*. *faecium* T-110, and *C*. *butyricum* TO-A, also increase faecal SCFA concentration in animals [67, 68]. Therefore, based on these results, we speculate that probiotics also increased SCFA production in the intestine in adenine-induced CKD rats in this study.

In this study, pH of faeces was elevated but the probiotics decreased it in rats with CKD caused by adenine. In the intestine, pH is elevated owing to the high ammonia levels in the gut during CKD [69]. David Ríos-Covián et al. [70] and Joanne Slavin [71] reported that SCFA lowered the intestinal pH, and in another study, probiotics lowered the intestinal pH in chickens by producing SCFA [68].

In this study, intestinal permeability increased in rats with CKD caused by adenine and the probiotics decreased it. Previous studies have shown increase in intestinal permeability in CKD [72]. In addition, Austin Gonzalez et al. [73] reported that sodium butyrate improved the intestinal permeability in CKD rats. In our study, faecal butyrate concentrations were increased at Week 4 in the probiotics group. Based on the results of previous studies, it was suggested that probiotics improved intestinal permeability in adenine-induced CKD rats by producing SCFA in the intestinal tract in this study.

In CKD, previous animal experiments have shown that increased intestinal permeability causes live bacteria to cross the intestinal barrier and migrate to the liver, resulting in increased levels of bacterial endotoxin in serum [72]. However, no correlation was identified between the endotoxin levels and CKD stages in other studies [41, 74]. Endotoxin detection may not be the best assay for analysing exposure to bacterial fragments due to the endotoxin’s brief half-life and limitations of the limulus amoebocyte lysate assay for endotoxin detection [72]. Therefore, in this study, we examined the concentration of sCD14 as a marker of host response to endotoxin exposure. CD14 plays a vital role as a pattern-recognition receptor for endotoxins in the generation of an innate immune response [41]. CD14 is either present as a soluble molecule (sCD14) following secretion or enzymatic cleavage, or it is membrane-bound with a 13 glycosylphosphatidylinositol anchor [41]. As an endotoxin receptor, sCD14 concentrations increase with loss in renal function and are linked to mortality [41]. In this study, the plasma sCD14 concentration increased in rats with CKD caused by adenine and probiotics decreased it. It was suggested that the increased intestinal permeability caused by adenine-induced CKD increased the transfer of gut-derived lipopolysaccharides (LPS) into the bloodstream, and this effect is alleviated by probiotics.

In this study, serum concentrations of seven uremic toxins were elevated in rats with CKD caused by adenine, while probiotics decreased them. It has been reported that the plasma uremic toxin levels increase with the progression of CKD [75]. The gut microbiota is responsible for the generation of uremic toxins [76–78], which accumulate in the blood circulation of patients with CKD [79–81]. Low intestinal pH reduces the activity of unwanted bacterial enzymes and accelerates the breakdown of peptides linked to the production of hazardous substances, including ammonia, amines, and phenolic compounds [71]. This study suggests that in adenine-induced CKD rats, probiotics lowered intestinal pH and reduced the production of urotoxins in the intestine while decreasing intestinal permeability and the transfer of bacterial components into the bloodstream.

In this study, the amounts of *Bifidobacterium* sp., *Lactobacillus/Enterococcus* spp., and *C*. *coccoides-E*. *rectale* in faeces decreased, and the amount of *E*. *coli* spp. increased in rats with CKD caused by adenine. However, probiotic treatment increased the amount of *Bifidobacterium* sp., *Lactobacillus/Enterococcus* spp., and *C*. *coccoides-E*. *rectale* in faeces and decreased the amount of *E*. *coli* in adenine-induced CKD rats. In general, *Bifidobacterium* sp. are known to activate intestinal immunity and produce butyric acid from lactic acid and acetic acid in the intestines, while *Lactobacillus/Enterococcus* spp. produce lactic acid in the intestines and inhibit pathogenic bacteria. *E*. *coli* and is also known to cause endotoxin shock when it is transferred from the intestinal tract to the bloodstream. It has been previously reported [75] that in CKD, the number of *Bifidobacterium* sp. and *Lactobacillus* spp. decreased and that of *E*. *coli* increased as the renal function declined. It was also reported that probiotics, including *B*. *subtilis* TO-A, *E*. *faecium* T-110, and *C*. *butyricum* TO-A, decreased the number of *E*. *coli* in faeces [82]. It was also reported that *B*. *subtilis* TO-A produces bifidobacteria growth factors in the intestine and increases its amount [83].

In this study, the serum BUN and Cre levels were elevated in adenine-induced CKD rats and the probiotics suppressed this elevation. The serum BUN and Cre levels are renal function markers and are known to increase during CKD progression. Indoxyl sulphate, a protein-bound indole uremic toxin that accumulates in CKD, causes tubular toxicity by directly inducing cell death through apoptosis or necrosis [84]. Indoxyl sulphate also increases oxidative stress and reduces antioxidant capacity, leading to tubular cell injury and inflammation of the interstitial fluid [84]. The injured renal tubule activates TGF1 signalling, drives interstitial inflammation and renal fibrosis in response to induction by Indoxyl sulfate, and is involved in the pathogenesis and progression of CKD [84]. Clinically, serum indoxyl sulphate concentrations are significantly higher in advanced CKD, and its value is a useful marker for predicting reduced renal function in patients with CKD [84]. Since uremic toxins can impair the renal function, it can be speculated that probiotics reduced the renal dysfunction by decreasing the blood uremic toxin levels in adenine-induced CKD rats in this study.

In this study, probiotics decreased the elevated serum P level in adenine-induced CKD rats. At all phases of CKD, hyperphosphataemia is a generally acknowledged risk factor for mortality and cardiovascular disease [85]. It was also reported that the blood P levels increased in adenine-induced CKD rats [86]. Bifidobacteria produce SCFA and makes the intestinal lumen more acidic and, therefore, enhances Ca ionisation. Ionised Ca binds free phosphate ions, resulting in reduced serum P levels [87].

In this study, muscle strength and function were attenuated in rats with CKD caused by adenine, and probiotics enhanced it. At Week 4, the muscle strength in the probiotics group was 1.03-fold higher than that in the control group and 0.92-fold higher than that in the normal group. At Week 4, muscle function in the probiotics group was 1.22-fold greater than that in the control and 0.90-fold greater than that in the normal group. To compare the findings of the present study with those of previous studies, we searched the literature for studies assessing the effects of probiotics on muscle strength and function in adenine-induced CKD rat models. However, to the best of our knowledge, no such studies have been published. Therefore, we compared the present study with previous studies assessing the effects of probiotics on muscle strength and muscle function in aged mice [88]. The effects of probiotics on muscle strength and muscle function improvement in the present study were weaker than those in previous studies. This may be attributed to the differences in the way sarcopenia is induced and differences in the duration of probiotic administration. According to Ono et al. [89], LPS blocks myogenic differentiation through the toll-like receptor 4-nuclear factor-B-dependent and autocrine/paracrine tumour necrosis factor-α-induced pathways, and these pathways may contribute to the onset of muscle wasting brought on by metabolic endotoxemia or sepsis. It was also reported that accumulation of uremic toxins triggers skeletal muscle loss or dysfunction in CKD [17]. Furthermore, probiotic treatment has been shown to decrease the serum endotoxin levels in infants [26]. Therefore, the inhibitory effect of probiotics in the present study on reduced muscle strength and muscle function may be related to improved intestinal permeability and reduced transfer of LPS and uremic toxins into the blood.

There are some limitations of this study. First, this study did not investigate uremic toxins in the gut. This is because no correlation exists between the uremic toxin concentrations in the intestine and blood, and there was no association between uremic toxin concentration in the intestine and CKD [75]. Second, we investigated the intestinal permeability, but not the barrier function, e.g. tight junction proteins and mucins. Since it has been reported that the intestinal barrier function decreased in CKD [72], further detailed investigation is needed. Third, this study only investigated a limited number of intestinal bacteria, and more widespread investigation using a more accurate technique, such as next-generation sequencing, is needed, as there are reports stating that various intestinal bacteria are affected in CKD [75]. Finally, this study was conducted for future human applications. Especially, it was conducted on the basis of doses approved for humans; therefore, different doses and dose-responses were not studied. The approved use of the probiotics used in this study is for ’various symptoms caused by abnormalities of the intestinal microbiota’. As a rule, physicians can only administer approved doses to patients and cannot guarantee their safety at doses unapproved. In addition, the probiotics used in this study were formulated as a mixture of three bacteria and administered to humans. Therefore, the effect of each bacterium alone has not been investigated. It will be necessary to investigate the dose-response and efficacy of the individual bacteria in the formulation in the future.

## Conclusion

In conclusion, our results indicate that safety-guaranteed probiotics, including *B*. *subtilis* TO-A, *E*. *faecium* T-110, and *C*. *butyricum* TO-A, improved SCFA production, intestinal permeability, pH of the intestine, intestinal microflora, blood renal function markers, and uremic toxins in blood in rats with CKD caused by adenine. The findings of this study show the potential of medical probiotics in preventing the progression of CKD, especially where safety is required. Further studies are needed to validate these findings in humans.
